# Effect of Weight Distribution and Active Safety Systems on Electric Vehicle Performance

**DOI:** 10.3390/s24113557

**Published:** 2024-05-31

**Authors:** Valerio Gori, Will Hendrix, Amritam Das, Zhiyong Sun

**Affiliations:** 1 Faculty of Mechanical Engineering, Delft University of Technology, 2628 CD Delft, The Netherlands; v.gori@student.tudelft.nl; 2 Department of Electrical Engineering, Eindhoven University of Technology, 5612 AZ Eindhoven, The Netherlands; w.h.a.hendrix@tue.nl (W.H.); am.das@tue.nl (A.D.)

**Keywords:** electric vehicle, weight distribution, active safety systems, wheel slip controller, torque vectoring

## Abstract

This paper describes control methods to improve electric vehicle performance in terms of handling, stability and cornering by adjusting the weight distribution and implementing control systems (e.g., wheel slip control, and yaw rate control). The vehicle is first simulated using the bicycle model to capture the dynamics. Then, a study on the effect of weight distribution on the driving behavior is conducted. The study is performed for three different weight configurations. Moreover, a yaw rate controller and a wheel slip controller are designed and implemented to improve the vehicle’s performance for cornering and longitudinal motion under the different loading conditions. The simulation through the bicycle model is compared to the experiments conducted on a rear-wheel driven radio-controlled (RC) electric vehicle. The paper shows how the wheel slip controller contributes to the stabilization of the vehicle, how the yaw rate controller reduces understeering, and how the location of the center of gravity (CoG) affects steering behavior. Lastly, an analysis of the combination of control systems for each weight transfer is conducted to determine the configuration with the highest performance regarding acceleration time, braking distance, and steering behavior.

## 1. Introduction

Electric vehicles (EVs) have increased in popularity due to their influence on reducing greenhouse gas emissions, reducing the impact of pollution on human health and hence contributing to a cleaner environment [1,2]. According to the market outlook, 58% of new car sales will be of electric vehicles by 2040 [3]. Due to the electrification in the automotive world, the possibility of the functionality of a vehicle has significantly been affected. Research aimed at improving the safety of vehicles to reduce car accident fatalities has increased substantially. An example is an active stability control system that prevents vehicles from spinning, drifting, and rolling over. The most commercialized stability control systems are based on differential braking and torque vectoring which apply a different braking or driving torque to each driving wheel to achieve the desired yaw moment, respectively. This can be achieved when the wheels are driven separately by two electric motors [4,5]. Moreover, electric vehicles, due to their architecture, offer greater potential compared to conventional vehicles regarding longitudinal motion.

EVs with individually driven wheels allow the development of control algorithms that can significantly improve vehicle performance through anti-lock braking system (ABS) and traction control (TC) [6]. Such systems are essential in vehicles as they assist the driver to keep the vehicle stable and follow the desired trajectory. The systems are based on the feedback control of the lateral dynamics parameters, such as the side-slip angle and yaw rate responses. Some researchers have focused solely either on the control of the yaw rate response to increase responsiveness to steering inputs or on the feedback control of the side slip angles to enhance stability; others attempted to combine both feedback controls to further increase vehicle stability performance [7]. To achieve this, various control algorithms have been developed and presented in the literature: for example, regarding the electronic stability control, PID, state feedback control, optimal control and sliding mode-based controllers have been used [8]. Das et al. proposed a modular hierarchical control architecture for multi-wheeled vehicles [9]. M. K. Aripin et al. evaluated a non-linear feedback algorithm and sliding mode for yaw rate control [10]. Benoit Lacroix et al. conducted a study to compare different methods on direct yaw moment control (i.e., PID and sliding mode) using a 2-DOF vehicle model [7]. Similar methods were implemented by Andoni Medina et al. who compared typical control methods used for ensuring vehicle stability and improving lap time for electric racing cars using PID and sliding controllers [11] as well as Leonardo De Novellis, et al. who analyzed and compared different PID and sliding mode-based control techniques (e.g., SOSM controllers) [12]. Gökhan Tekin et al. developed a fuzzy logic control scheme for active yaw rate and side slip angles feedback control [13]. Haiping Du et al. analyzed the yaw rate and side slip angle responses of a vehicle when applying a controller based on a finite numbers of linear matrix inequalities (LMIs) [14]. Alberto Parra et al. presented a study on nonlinear model predictive controller on EV with multiple drive trains to enhance energy efficiency through the control of the cornering performance [15]. Last, A. Parra et al. and Q. Lu et al. proposed intelligent and $H_{\infty }$ controllers, respectively [16,17].

Furthermore, weight distribution is a key parameter in road vehicle design as different loading conditions may lead to more aggressive under- or oversteering behavior aggravating the stability of a vehicle. Weight distribution also influences the maximum force that can be transmitted to the wheels. Research has been conducted to analyze the dependency of weight distribution on the driving behavior. Ekalak Prompakdee et al. conducted a research aimed at studying the relation of the understeer gradient with the weight distributions on intercity buses under steady state conditions [18]. The driving performances under various loading conditions have also been analyzed in [19] to study the effect on the braking distance in road freight transport. An analysis on weight distribution aimed at maximizing the cornering speed of formula cars has also been conducted by H. Nozaki [20]. Lastly, a lot of research has been ongoing for developing anti-lock braking systems and traction controllers: often, such systems are based on wheel slip controllers; however, various methods have been investigated. Regarding the control schemes, PID is most often used [21]. For example, Min et al. show the performance of PID and fuzzy controller on ABS development [22]. Taketoshi Kawabe et al. developed a wheel slip controller based on sliding mode for commercial vehicles on low friction roads [23]. Ma et al. evaluated the performance of wheel slip controller based on model predictive control considering road roughness and low adhesion surfaces [24]. Dzmitry Savitski et al. compared PI, first-order sliding mode, integral sliding mode and continuous twisting algorithms applied to a wheel slip controller on fully electric vehicles [25].

This research combines the implementation of a yaw rate controller and a wheel slip controller to improve longitudinal and cornering performance to different weight distributions in order to determine the configuration with the highest performance in terms of safety, handling and stability. To conclude, the literature presents studies on the implementation of effective electronic stability controls, on wheel slip controllers for ABS or TC and also on the effect of loading condition on driving behavior for different vehicles; however, a study combining the weight distribution analysis and the implementation of control system algorithms to fully improve performance on rear-wheel driving electric vehicles is missing. In other words, the article is aimed at showing how weight distribution and control systems can be designed and combined to improve the driving behavior.

This paper is organized as follows: Section 2 shows the experimental set up and the methods used to model the vehicle and implement the control schemes. Then the results are shown and discussed in Section 3 and Section 4, respectively. Finally, some concluding remarks are made in Section 5. A video on the results obtained can be seen at the following url: https://youtu.be/wRzeLYJABbQ (accessed on 25 May 2024).

## 2. Materials and Methods

### 2.1. Experimental Set Up

**Figure 1:**
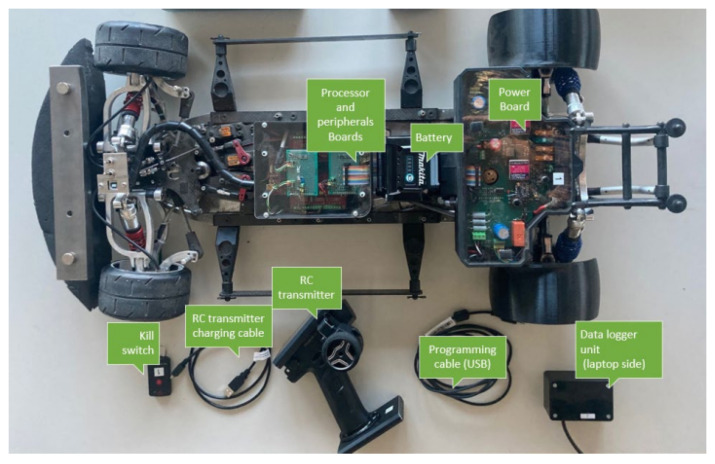
Experimental set up: vehicle system.

**Figure 2:**
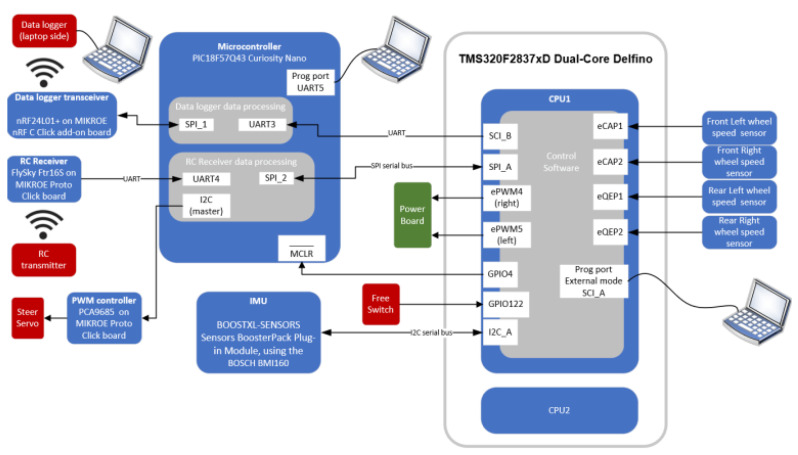
Experimental set up: software system overview.

The RC car used to perform the experiments is an FG Competition EVO 08-510 (FG Modellsport GmbH, Winterbach, Germany). The vehicle is in scale 1:5; about five times smaller than a real car. The main components can be seen in Figure 1 and an overview on the processor boards and their connections is shown in Figure 2. The vehicle originally had an internal combustion engine which had been replaced by two electric motors, each driving the rear wheel through a gearbox designed in-house at the Eindhoven University of Technology. The vehicle is driven by a remote controller which sends the steering and throttle percentage to the receiver on board. The steering percentage is directly sent to the front tires servos, while the throttle percentage is first sent to the DSP. By implementing control schemes on the DSP it is possible to actively control the torque that is delivered to each driving wheel. The car originally had friction brakes; however, they have been disconnected and the vehicle is braked through the electric motors on the rear tires. The motors are three-phase synchronous machines with a maximum output power of 419 W corresponding to a maximum torque of 5.29 Nm delivered to each driving tire. In case of emergency, the motor can be short-circuited through a resistive circuit. To power the vehicle, a Makita 40 V, 4 Ah Li-ion battery pack is used. For safety reason a battery management system is implemented: if the battery voltage drops below 3 Volts, the motors are deactivated and short-circuited.

**Figure 3:**
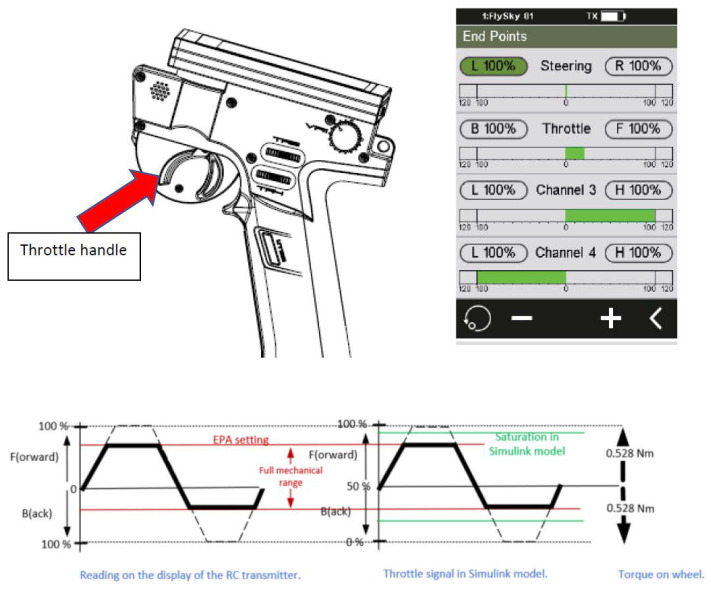
Experimental set up: throttle handle and torque generation.

The torque setpoints for the left and right rear motor (from the control actions) are sent using PWM signals to the power board electronics. The full mechanical range of the throttle handle on the RC transmitter is mapped to a maximum of 0–100% value in the forward driving direction (pulling the handle) and 0–100% in the backward driving direction (pushing the handle). The reading on the display shows the current value. This mapping of the mechanical range is set by the End Point Adjustment (EPA) value. The throttle handle and torque generation are shown in Figure 3.

The DSP is equipped with a Texas Instruments (TI)eZDSP F28355 board (Dallas, TX, USA) and AMBER wireless data transfer system (Trier, Germany) that allows the data to be transferred to a PC with a frequency of 200 Hz through the data logger unit. The board is supported by Matlab embedded encoder. Hence, the control schemes can be directly implemented on Matlab and Simulink 2021b [26]. The program is implemented and uploaded on the vehicle through the USB programming cable. Last, the vehicle is equipped with the following sensors:

- wheel speed sensors on each tire
- gyroscope

The wheel speed sensors are located on the inside of each wheel. They consist of six black and six white stripes of film located along the circumference in the inside of the wheel. As the wheel rotates, the transition of the black and white regions are detected by CNY70 optical sensors located on a PCB inside the wheel. The velocity is calculated from the time elapsed between each transition. The gyroscope is an MPU-6000 (InvenSense Inc., Sunnyvale, CA, USA) that calculates the rotation around the z-axis (i.e., the yaw rate). To conclude, the data gathered from the gyroscope and speed sensor allow for the implementation of feedback to individually control the torque in each motor.

### 2.2. Vehicle Dynamics Modeling

**Figure 4:**
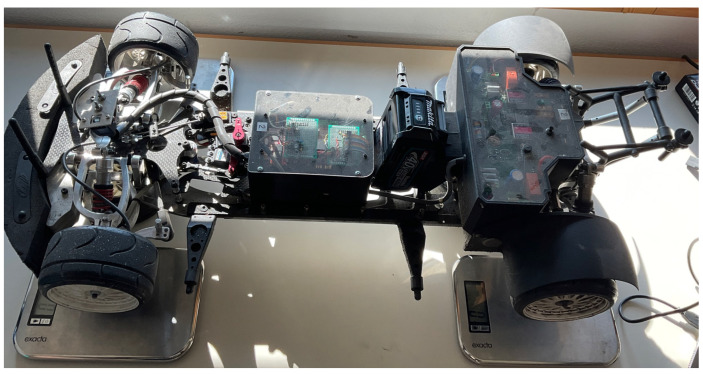
Vehicle on scales for weight measurement.

The main factor for analyzing the weight distribution of a vehicle is the position of its center of gravity (CoG). Determining the CoG involves weighing the car. As depicted in Figure 4, each tire is precisely positioned on the center point of a scale to measure its weight. Furthermore, the vehicle configuration ensures that the Center of Gravity (CoG) is equidistant from the left and right wheels. It results in equal forces being experienced by the left and right tires.

Next, given that the car is standstill, the following equations of motion are derived:

(1) $$\left\{\begin{array}{c} I_{yy}{\dot{\omega }}_{y}=F_{z1}a-F_{z2}b=0\\ l=a+b\\ mg=F_{z1}+F_{z2} \end{array}\right.$$

where $F_{z1}$ and $F_{z2}$ are the normal forces acting on the front and rear tire, respectively, obtained by the measurements conducted as in Figure 4. $I_{yy}$ is the pitch mass moment of inertia, $\dot{\omega_{y}}$ is the pitch acceleration, *l* is the wheelbase and *m* is the mass. Solving the equations above for each weight configuration allows to derive the corresponding *a* and *b* values representing the distance between the CoG and the front and rear tires, respectively, as shown below:

(2) $$\left\{\begin{array}{c} a=\frac{F_{z2}}{mg}l\\ b=\frac{F_{z1}}{mg}l. \end{array}\right.$$

**Figure 5:**
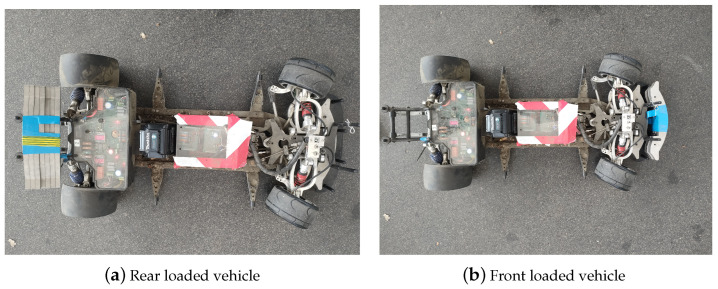
Vehicle loading condition.

In order to investigate various loading conditions, weights were placed on the front and on the rear of the vehicle to achieve different CoG locations. More specifically, the weights have been attached to the tail (Figure 5a) and to the front bumper (Figure 5b) to maximize the change in weight. The set up for the testing vehicle with front- and rear-loaded weights is illustrated in Figure 5. The experiments were conducted on these two configurations as they represent the most extreme loading condition possible for the vehicle in consideration. In order to validate the yaw rate and wheel slip controllers, the tests were conducted on the unloaded vehicle shown in Figure 4.

### 2.3. Vehicle Model

This section deals with the differential equation used to describe and model the vehicle behavior. For the scope of this research, the bicycle model has been used due to its simplicity, computational efficiency, and accuracy related to the control objective.

The bicycle model consists of the longitudinal (*u*), lateral (*v*) and yaw motion (*r*) as shown in ([27] Figure 1.9) which shows the global coordinates denoted as X and Y, the lateral and longitudinal directions, *u* and *v*, together with the yaw rate (*r*) moments denoted as $M_{z}$ and the parameters a,b,l. Moreover, the model is based on the following assumptions:

- The left and right tires are lumped together in one equivalent tire.
- Pitch and roll are not taken into account: the height of the center of gravity is assumed to be zero.
- The vehicle is assumed to drive on a flat surface.

We remark that these assumptions are reasonable in practice. The first assumption is for deriving a bicycle model; the second assumption is based on the bicycle dynamics (in a 2-D space); the third assumption is according to the actual testing environment. In order to model the vehicle, two reference frames are used, i.e., the global or ground reference frame (X,Y) and the body reference frame frame (i.e., the one relative to the vehicle direction), denoted by (*u*,*v*,*r*) where the *u*-axis is the the longitudinal axis of the vehicle. The origin of the body frame is given by the center of gravity [28]. The subscript *x* and *y* are used to indicate the longitudinal and lateral directions, respectively. Next, the forces and moments acting on the rear-wheel driven vehicle are determined and the following equations are derived to describe the vehicle behavior in the body frame ([27]).

(3) $$\left\{\begin{array}{cc} F_{u} & =F_{x2}-\sin\left(\delta \right)F_{y1}-F_{d}\\ F_{v} & =F_{y2}+\cos\left(\delta \right)F_{y1}\\ M_{z} & =M_{tv}+a\left(\cos\left(\delta \right)\right)F_{y1}-bF_{y2}. \end{array}\right.$$

where $M_{z}$ denotes the yaw moment around the z-axis. For simplicity, this model only considers the yaw moment around the z-axis denoted as $M_{z}$ rather than the moments around each tire. The model also takes into account the drag forces $F_{d}$ ($F_{d}$ is a sum of rolling resistance and air resistance forces.) Moreover, since the test vehicle is RWD, the longitudinal force on the rear tires is equal to zero. The relation between torque and throttle is assumed to be linear, hence the force applied to the driving wheel $F_{x2}$ is related to the input torque through the wheel radius $r_{w}$. The vehicle drag forces are given as a combination of rolling resistance, and aerodynamic drag. For the scope of this research, such forces have not been measured individually, but the combined resistance force denoted by $F_{d}$ is calculated experimentally from a coast-down test. $M_{tv}$ represents the extra moment due to torque vectoring, and is determined by the following equation:

(4) $$M_{tv}=\frac{\Delta T}{r_{w}}w,$$

where ΔT is the torque difference applied to the wheels by the yaw rate controller, e.g., the output of the controller. *w* is the vehicle width. Furthermore, the vehicle trajectory in terms of the global coordinate systems is derived as follows:

(5) $$\left\{\begin{array}{c} \dot{X}=u\cos\left(\psi \right)-v\sin\left(\psi \right)\\ \dot{Y}=u\sin\left(\psi \right)+v\cos\left(\psi \right)\\ \dot{\psi }=r, \end{array}\right.$$

where ψ is the angle between the body and the global reference frame, and *r* denotes the angular velocity. Combining Equations (3) and (5) leads to the equations of motion according to Newton’s laws:

(6) $$\left\{\begin{array}{c} m\dot{X}=F_{u}\cos\left(\psi \right)-F_{v}\sin\left(\psi \right)\\ m\dot{Y}=F_{u}\sin\left(\psi \right)+F_{v}\cos\left(\psi \right)\\ I_{zz}\dot{r}=M_{z}, \end{array}\right.$$

where $I_{zz}$ is yaw moment of inertia. After taking the derivative with respect to time for Equation (5) and substituting it in Equation (6), the vehicle model below is found:

(7) $$\left\{\begin{array}{c} m\left(\dot{u}-vr\right)=F_{u}\\ m\left(\dot{v}+ur\right)=F_{v}\\ I_{zz}\dot{r}=M_{z}. \end{array}\right.$$

### 2.4. Tire Lateral Dynamics and Steering Behavior

The tire is under the effect of a vertical load and a lateral force when turning which contributes to the vehicle heading angle. The tire lateral forces ($F_{y1}$, $F_{y2}$) have a non-linear relation with the side slip angles. However, by keeping the angles small, ($\alpha_{1}$ and $\alpha_{2}$, respectively) the lateral forces are assumed to be linearly proportional to the side slip angles. Such an angle is defined as the angle between the tire orientation and its velocity vector. The front and rear side slip angles are given in Equation (8), respectively.

(8) $$\left\{\begin{array}{c} \alpha_{1}=\delta -\arctan\frac{\left(v+ar\right)}{u}\\ \alpha_{2}=\arctan\frac{\left(v-br\right)}{u}. \end{array}\right.$$

Assuming linear tire behavior, the side slip angles are related to the lateral forces through the cornering stiffness ($C_{1}$ and $C_{2}$ for the front and the rear tires, respectively). The lateral forces can therefore be written as [5]:

(9) $$\left\{\begin{array}{c} F_{y1}\approx \alpha_{1}C_{1}\\ F_{y2}\approx \alpha_{2}C_{2}. \end{array}\right.$$

Last, an indicator of the vehicle cornering behavior is the understeer gradient η. It indicates the path curvature of the vehicle that results from a given steering angle δ at any speed. Given that the steering angle is expressed as the combination of the kinematic steering angle and the additional angle due to the lateral acceleration ($a_{y}$), δ can be written as:

(10) $$\delta =\frac{l}{R}+\eta \frac{a_{y}}{g}=\frac{l}{R}+\alpha_{1}-\alpha_{2}$$

where *R* is the turning radius, $a_{y}$ the lateral acceleration, *g* the gravitational acceleration, and η the under-steer gradient. Expressing the side slip angles as a function of the vertical forces allows us to express the steering angle as a function of lateral acceleration and static vertical load as shown below:

(11) $$\delta =\frac{l}{R}+\frac{a_{y}}{g}\left(\frac{F_{z1}}{C_{1}}-\frac{F_{z2}}{C_{2}}\right).$$

The under-steer gradient is given as:

(12) $$\eta =\left(\frac{F_{z1}}{C_{1}}-\frac{F_{z2}}{C_{2}}\right).$$

Note that dynamic load transfer should not be taken into account in this equations, hence the vertical forces $F_{z1}$ and $F_{z2}$ represent the static weight distribution. The driving behavior is related to the previous equations according to the following relation:

(13) $$\left\{\begin{array}{cc} \eta =0 & “\mathrm{neutral}\;\mathrm{steer}”\;\mathrm{for}\;\alpha_{1}=\alpha_{2}\\ \eta >0 & “\mathrm{under}\;\mathrm{steer}”\;\mathrm{for}\;\alpha_{1}>\alpha_{2}\\ \eta <0 & “\mathrm{over}\;\mathrm{steer}”\;\mathrm{for}\;\alpha_{1}<\alpha_{2} \end{array}\right.$$

In other words, to maintain a constant cornering radius *R* the steering angle has to increase for an understeered vehicle, decrease for an oversteered one and remain the same for a neutral steered vehicle [27].

The equations described in this section have been implemented in Matlab and Simulink to model the vehicle, given a torque and steering percentage as inputs. The data to validate the model have been gathered from a constant cornering experiment. In other words, the vehicle was driven along a constant radius with slowly increasing throttle. Then, the model was fitted to the measurement data by tuning the cornering stiffness values.

### 2.5. Yaw Rate Controller Design

The following paragraph deals with the implementation of a torque vectoring algorithm. In this research, a PID-type controller is applied due to its practicality, simplicity and effectiveness compared to other control methods [8,29]. However, due to the high frequency noise in the sensors, a PI controller is chosen. The controller designed in this paper is designed to impose a certain yaw rate to the vehicle. In other words, it is designed to minimize the error between the vehicle measured yaw rate and the reference (i.e., corresponding yaw rate for neutral steering condition) by redistributing the torque to the driving wheels. The reference yaw rate is calculated according to the small slip angle approximation. Given that at steady state the expression for path curvature under constant speed and steering angle is given below [27]:

(14) $$\frac{1}{R}=\frac{r}{V}\approx \frac{r}{u},$$

and that under kinematic steering, the steering angle is related to the turning radius through the following equation:

(15) $$\delta =\frac{l}{R},$$

substituting Equation (15) into Equation (14), yields the following reference:

(16) $$r_{ref}=u\left(\frac{\delta }{a+b}\right)$$

The longitudinal speed *u* is assumed to be the average velocity of the rear wheels assuming that they are not spinning nor they are locked. Hence, through feedback, the error is reduced by the PI action as follows:

(17) $$\Delta T=K_{p}e+K_{I}\int e,$$

where the error *e* is given as the difference between the reference $r_{ref}$ and measured yaw rate *r* as follows:

(18) $$e=r_{ref}-r,$$

**Figure 6:**
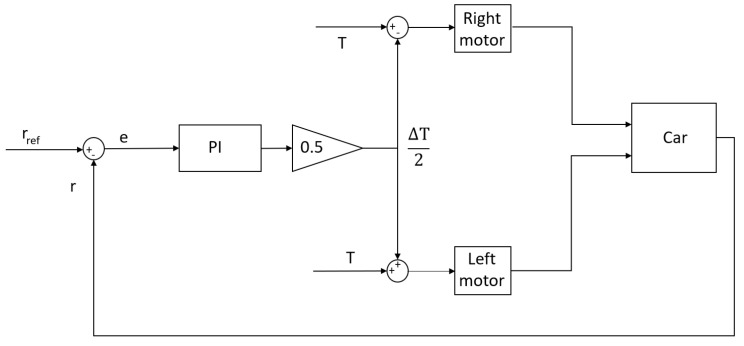
Yaw rate controller scheme.

Next, the torque (i.e., the output of the controller) is distributed between the left ($T_{left}$) and right motor ($T_{right}$) as follows:

(19) $$\left\{\begin{array}{c} T_{right}=T-\frac{\Delta T}{2}\\ T_{left}=T+\frac{\Delta T}{2} \end{array}\right.$$

where *T* is the input torque. Equation (19) is valid according to the following sign convention: turning clockwise is positive and anti-clockwise negative. The schematic of the controller is shown in Figure 6.

The parameters are obtained by manual tuning of the proportional $K_{P}$ and integral $K_{I}$ gains. The resulting values are displayed in Table 1.

### 2.6. Longitudinal Dynamics: Slip Controller

In order to improve the longitudinal performance of the vehicle, a wheel slip controller is implemented using a PI controller. The PI-type controller is implemented due to its effectiveness and simplicity [21]. A quarter car model consisting of a single wheel attached to a mass is used. According to this model, only longitudinal dynamics are considered; moreover, one of the limitations is the assumption of a fixed load on the wheel. The model and the underlying equations are shown in Equation (20).

(20) $$\left\{\begin{array}{cc} m\dot{v} & =-F_{x}\\ J\dot{\omega } & =r_{w}F_{x}+T\\ F_{x} & =F_{z}\mu \left(\kappa \right), \end{array}\right.$$

where *m* is the mass, $\dot{v}$ is the rate of change of velocity, *J* the wheel inertia, $F_{x}$ is the longitudinal tire force, $F_{z}$ is the normal force, *T* is the torque, $\dot{\omega }$ is the rate of change of the angular velocity and μ is the tyre road friction coefficients dependent on the slip ratio κ. By looking at the equation above it follows that, given a fixed vertical load, the value of the slip that leads to the highest friction coefficient must be found such that the maximum braking/traction force can be developed. The slip ratio is defined as the normalized difference between the vehicle velocity and the wheel velocity as follows [30]:

(21) $$\kappa =-\frac{v-\omega r}{v}.$$

Hence, the following relation holds

(22) $$\left\{\begin{array}{cc} \kappa =0 & \mathrm{free}\;\mathrm{rolling}\;\mathrm{tyre}\\ \kappa >0 & \mathrm{driving}\;\mathrm{tyre}\;\\ \kappa <0 & \mathrm{braking}\;\mathrm{tyre}\\ \kappa =-1 & \mathrm{locked}\;\mathrm{wheel} \end{array}\right.$$

Since the slip of a free rolling wheel (i.e., non-driving wheel), is equal to zero, the wheel slip of a rear-wheel driven car can be approximated as follows:

(23) $$\kappa \approx -\frac{\omega_{f}-\omega_{r}}{\omega_{f}},$$

which can be rewritten as:

(24) $$\omega_{r}\approx \omega_{f}\left(1+\kappa \right).$$

**Figure 7:**
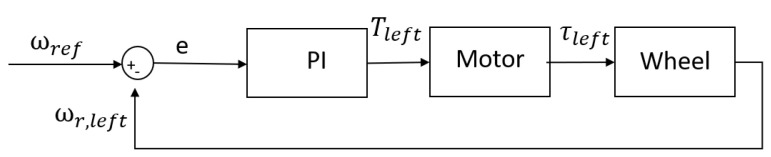
The control loop in the wheel model.

Since at very low speed ($\omega_{r}\approx 0$) the slip value is not defined, the slip is achieved by upper bounding the speed of the rear wheel as a function of the front tire as shown in Equation (24). Next, a PI controller is designed such that the wheel slip is kept constant at the point where the maximum force can be developed. The controller takes the difference between the measured rear wheel speed and the reference ($\omega_{f}\left(1+\kappa \right)$) and minimizes the error according to Equation (17). In this case, the error is given as the difference between the rear tire velocity and the reference. The torque (e.g., output of the PI controller) is added to the input torque such that the error is minimized. Figure 7 shows the control scheme for the left tire only as the scheme is equivalent for both tires. *T* indicates the input torque (e.g., output of the controller), τ is the output torque (e.g., torque applied to the tire).

Last, the value of the slip is set to be positive when the driver is accelerating, and negative when braking according to Equation (22), such that both anti-lock braking system (ABS) and traction control (TC) systems are obtained. Given that no data on the tire characteristics are provided, the ideal slip value has been determined experimentally through a braking and acceleration test. Regarding controller design, the gains are obtained manually tuning the controller. The resulting values are displayed in Table 2.

## 3. Results

The following section deals with the results gathered throughout the research. First, the vehicle driving performance is analyzed and the model is validated. Next, the yaw rate and the wheel slip controllers are evaluated. Then, the weight transfer analysis is conducted. At last, the combination of control algorithms and weight transfer is analyzed.

### 3.1. Steering Behavior and Model Validation

**Figure 8:**
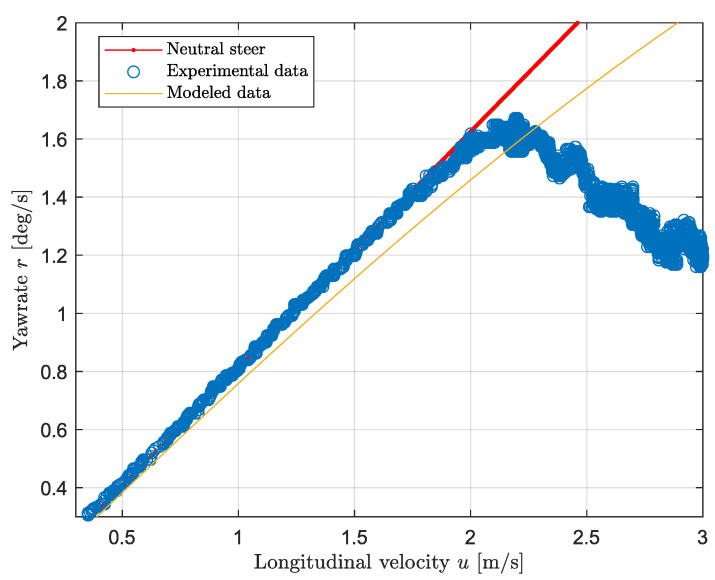
Vehicle steering behavior: modeled and experimental data.

In order to determine the unknown values of the cornering stiffness, a constant cornering test is conducted. This consists of steady state driving with constant steering angle for different velocities ranging from 0 to 3 m/s. Higher velocities were not reached due to the tire friction limits. The steering angle used throughout the experiments corresponds to 24.3 degrees. By looking at Figure 8, it is possible to compare the vehicle behavior with respect to the model and the reference values. Initially, the vehicle’s yaw rate follows the neutral steer reference, then at approximately 2 m/s, the vehicle starts to under-steer as the yaw rate drops. In the linear region, the discrepancy between the model and the measurement data is negligible; however, as the non-linear region approaches, the error reaches a maximum of about 9% at approximately 1.7 m/s. In the non-linear region, where the yaw rate suddenly drops, the error suddenly increments linearly with the speed: this is due to the non-linearities of the tires that are not taken into account by the bicycle model. In order to validate the model, the cornering stiffness values have been tuned manually to minimize the discrepancy. Given that the car is understeering and approximately 60% of the weight is on the rear, solving Equation (11) for a negative understeer gradient leads to the relation of $C_{1}<0.7$$C_{2}$. As a result, the modeled value of the front cornering stiffness is equal to 0.55$C_{2}$. More specifically the modeled value for the rear cornering stiffness is equal to 350 $\frac{N}{\mathrm{rad}/s}$, while the front value is equal to 192.5 $\frac{N}{\mathrm{rad}/s}$. The load configuration for this measurement is displayed by the “unloaded” case in Table 3.

### 3.2. Yaw Rate Controller

**Figure 9:**
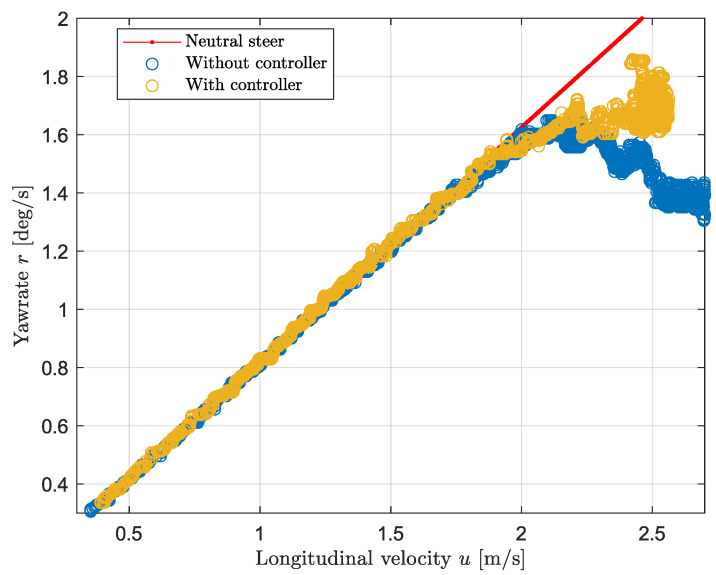
Vehicle steering behavior with yaw rate controller.

The results in this section are obtained by a steady state cornering test with a steering angle of 24.3 degrees. Figure 9 shows the vehicle behavior with and without the yaw rate controller. It follows that in the linear tire region, the controller does not contribute to the yaw moment as the vehicle is in neutral steering condition; however, in the non-linear tire region, a positive moment is applied to the vehicle improving its performance. In other words, the controller reduces the error by causing an increase in the yaw rate of approximately 15% at 2.5 m/s. Last, it is possible to notice that above a longitudinal speed of 2.5 m/s, the tires exceed their limits, where the input force exceeds the maximum force that can be developed by the tire. As a consequence, the vehicle starts to slide sideways in the direction of the turn. This can be seen by the sudden increase of the yaw rate which instantly reaches a maximum of 1.8 deg/s between 2.5 and 2.6 m/s.

### 3.3. Weight Transfer Analysis

**Figure 10:**
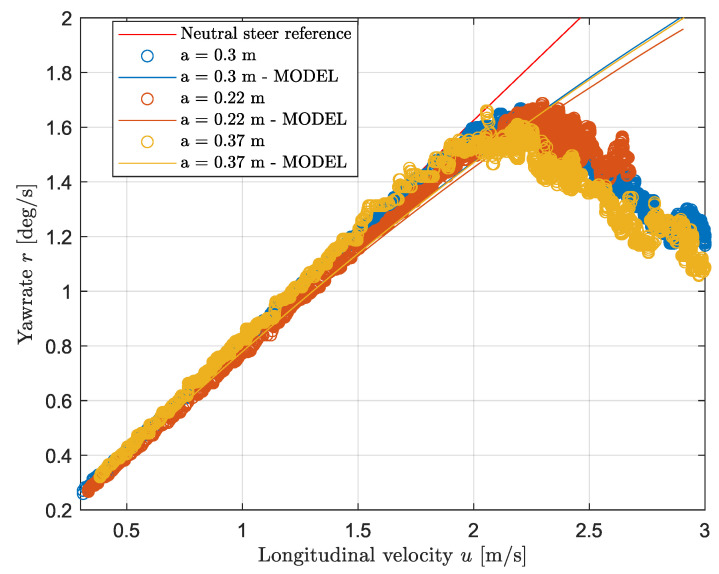
Vehicle steering behavior with yaw rate controller.

This subsection deals with the weight transfer analysis. Figure 10 shows the yaw rate as a function of velocity for both the modeled and the experimental data together with the reference for three weight configurations without applying any control systems. The loading conditions for this experiment are shown in Table 3, namely the case for a=0.37 corresponds to the “Loaded rear” case and a=22 is the “Loaded front” case. By looking at the figure, it follows that generally, for any weight configuration, the car shows understeering behavior at high speed as the yaw rate drastically drops at approximately 2.2 m/s. Next, both configurations for a=0.3 and a=0.37 show similar behavior at low speed: both cases show neutral steer behavior until the non-linear tire region (u> 2 m/s) is entered, whereas the third configuration shows a higher mismatch with the reference. More specifically, when the CoG is moved forward, the vehicle starts to understeer at a lower speed: the error between the reference and the measured data reaches a value greater than 5% already at a velocity of 1 m/s, then it increases linearly with the speed. Regarding the peak values, shifting the CoG forward leads to a slightly higher maximum yaw rate achievable compared to the other cases, namely a 5% increase in the maximum yaw rate is achieved by shifting the CoG to the front by 16 cm. However, the peak value is reached at a slightly higher speed. Last, in the non-linear region, the configuration for a=0.37 m shows the lowest yaw rate for any given velocity, while moving the CoG forward leads to higher yaw rate values. More specifically, moving the CoG rearward by 15 cm leads to a decrease of the yaw rate approximately by 15% in the high speed region. To conclude, loading the front axle leads to a more severe understeering in the linear region while loading the rear axle reduces such behavior. However, the opposite phenomena appears at high speed as the lowest yaw rate is achieved when loading the rear axle. Furthermore, Figure 10 shows that the model matches the observation above; however, a mismatch with regard to the experimental data appears at high speed due to the linear nature of the model.

### 3.4. Wheel Slip Controller

**Figure 11:**
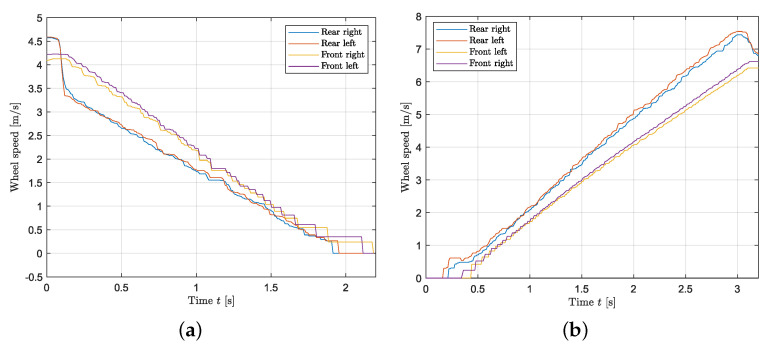
Wheel slip controller: wheel velocity against time. (**a**) Wheel slip controller during braking maneuver (ABS). (**b**) Wheel slip controller during acceleration manoeuvre (TC).

**Figure 12:**
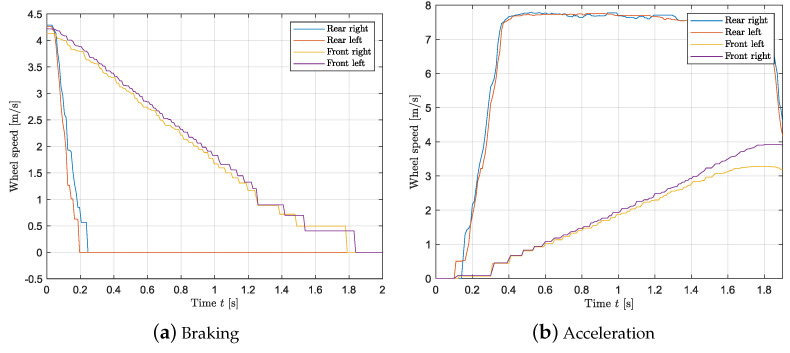
Wheel speed during a launch and braking maneuver with no slip controller.

**Figure 13:**
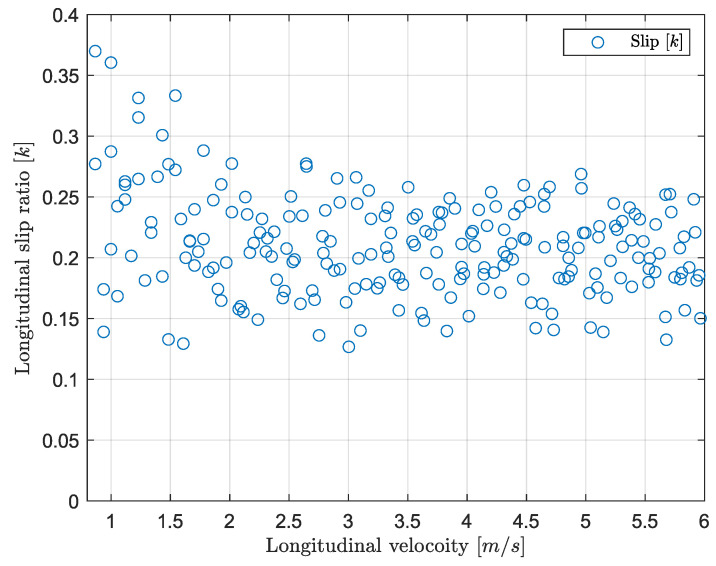
Values of slip: launch control.

**Figure 14:**
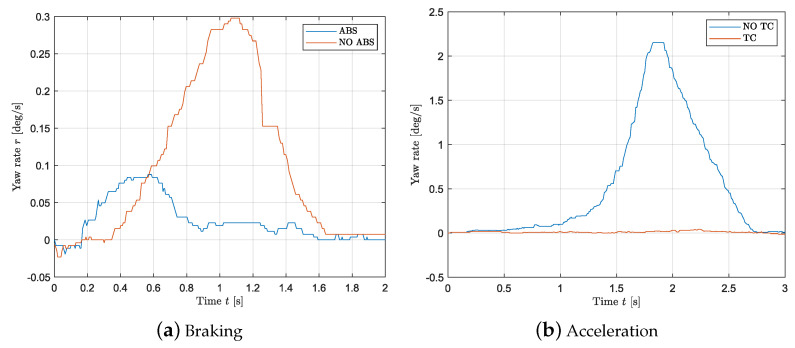
Vehicle speed during a launch and braking maneuver with no slip controller.

This paragraph deals with the validation of the slip controller. Figure 11a,b show the resulting measurements while braking and accelerating when the vehicle is unloaded, respectively. In order to evaluate the controller performance, a step of −2.5 Nm and 2.64 Nm is applied for braking and launching without applying a steering input, respectively. The loading condition corresponds to the “unloaded” case shown in Table 3. By looking at Figure 11a,b, it follows that the wheel slip controller prevents the driving wheels from locking and spinning as in Figure 12a,b. Figure 13 shows the values of the slip as the vehicle accelerates when the controller is applied as a function of velocity. It follows that constant slip of approximately 0.2 is achieved at all speeds. Since the wheels spin when the controller is not applied, it is not possible to calculate a reasonable value of slip using the methods above; however, Figure 12b shows that a very high difference in speed between the front and rear tires is achieved suggesting a high slip value. Hence, the wheel slip controller allows the vehicle to maximize the driving/braking force (see Equation (20)). An average deceleration of 2 m/s^2^ is achieved while braking, and an average acceleration of 2.3 m/s^2^ is reached when launching. Last, with a slip controller, the stability of the vehicle is highly increased as any unwanted yaw moment is rejected: Figure 14a,b show that when the wheel slip controller is not applied, the vehicle loses stability as a yaw moment is generated. Due to the nature of the experimental set up, the values of velocity and acceleration are given as an indication based on the testing results; they would be used to represent reasonable real-life values (after a scaling) for large-scale vehicles.

### 3.5. Control Algorithms for Different Weight Configurations

**Figure 15:**
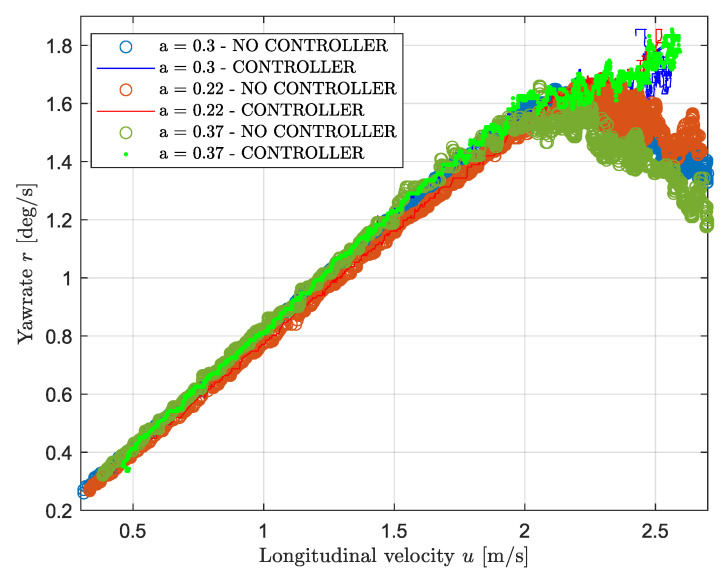
Weight transfer analysis: yaw rate against longitudinal velocity with a yaw rate controller.

The loading conditions shown in Table 3 have been implemented for testing the vehicle performance for different weight configurations. In addition, the experiments have been performed with the yaw rate and wheel slip controllers. Figure 15 shows the yaw rate value as a function of the longitudinal velocity. By looking at the Figure, it follows that the same observation as in Section 3.2 can be gathered. In other words, implementing the controller improves the vehicle performance in the non-linear region of the tires for every loading condition. Moreover, it follows that approximately the same performance can be achieved for each configuration. Therefore, given that in the non-linear region, the configuration with the CoG moved rearward (a=0.37) has the lowest angular velocity, the most significant improvement can be seen in this configuration as the yaw rate is increased by approximately 30% at 2.5 m/s.

**Figure 16:**
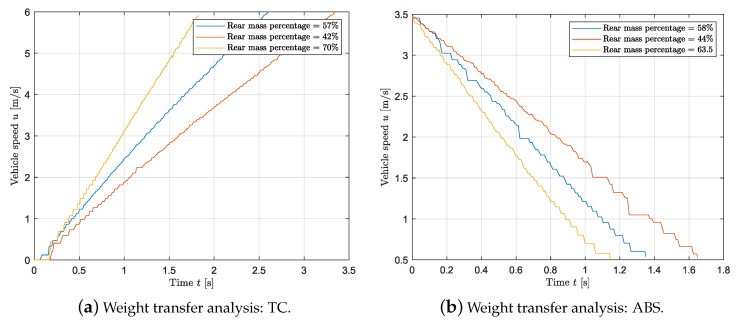
Weight transfer analysis: wheel slip controller.

**Figure 17:**
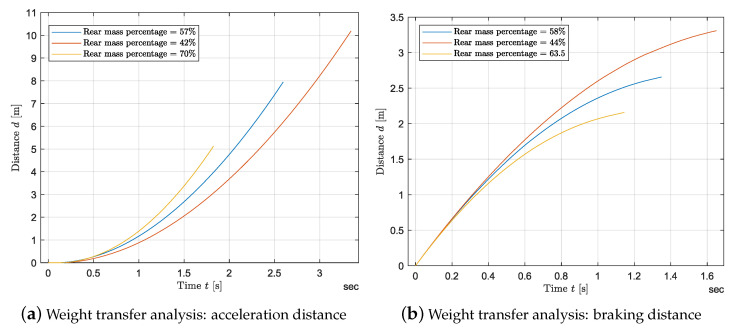
Braking and accelerating distance for different weight distribution.

Last, an analysis of the vehicle’s longitudinal performance with a wheel slip controller for each weight configuration is conducted. Figure 16a and Figure 17a show the longitudinal velocity of the vehicle as a function of time while accelerating and braking for the three loading conditions, respectively. To evaluate the performance while accelerating, the vehicle has been loaded as in Table 3. The loading conditions throughout the braking tests are displayed in Table 4. The same inputs as in Section 3.4 are applied. From Figure 16, it follows that by shifting the CoG rearward, hence increasing the static vertical load on the rear axle, the vehicle shows a higher acceleration. Namely, for the braking case, increasing the rear weight distribution from 42% to 70%, leads to an increase of 48% in the average acceleration. According to Figure 17a, there is a linear relation between the maximum force developed by the tire and the vertical static force on the driving axle. Figure 16b shows the vehicle velocity for a launch with different weight configurations. The same conclusion as for Figure 17a can be drawn. The acceleration linearly increases with the vertical load on the rear axle. In this case, increasing the rear vertical load by 43% leads to an increase in acceleration from 1.78 m/s^2^ to 3.2 m/s^2^. As a consequence the braking and the acceleration distance are highly reduced when the CoG is shifted to the rear or the rear axle is loaded. Namely for the acceleration, increasing the rear mass percentage from 42% to 70% leads to a 50% decrease in the acceleration distance, while the braking distance is reduced by approximately 34% when the mass percentage on rear is shifted from 44% to 63.5%

## 4. Discussion

First, the research shows that, given the assumptions in Section 2.2, the bicycle model captures the essential dynamics of the vehicle especially in the linear tire region, hence it can be considered an accurate model in mimicking the cornering behavior of the vehicle. The bicycle model allows to design and implement an effective yaw rate controller. However, the bicycle model also presents some limitations as it is based on several assumptions as detailed in Section 2. Hence, a different model may be used to examine the impact of the driver or of passengers. Moreover, the model assumes the height of the center of gravity to be zero. In general, EVs are heavier compared to the corresponding vehicles with ICE [31], so this is a reasonable assumption. Moreover, the load transfer is neglected which ensures the accuracy of the model at low lateral and longitudinal acceleration. As for severe maneuvers that produce large lateral accelerations, the bicycle model does not represent the vehicle response accurately due to nonlinear tire forces and associated dynamic load transfer. In other words, a different approach is suggested to model such maneuvers.

As shown in Figure 9, the cornering performance has improved as the yaw rate has increased up to 30% when the CoG is shifted to the rear. However, the effect of the controller is limited by the vehicle physical limits. When the force applied to the wheel exceeds the friction circle, the tires start spinning, hence the vehicle loses stability. Implementing a slip controller to prevent the wheel from spinning or locking while cornering will further improve the vehicle’s performance. Regarding the longitudinal motion, applying a wheel slip controller prevents the wheel from spinning and from locking, enhancing performance with regard to the braking distance and acceleration time and it greatly improves vehicle stability as any unwanted moment is completely rejected. However, throughout the experiments the same tires have been fitted to the vehicle and the tests were performed on a flat surface with a constant tyre road coefficient. The paper [32] suggest that to ensure the effectiveness of the control system, information of the peak tyre–road friction coefficient and adjustment of the slip ratios are fundamental. The implementation of a control algorithm based on the estimation of the tyre road friction may be beneficial in practice.

Figure 10 shows the importance of the weight distribution in vehicles regarding cornering performance. The relation between the weight distribution and the driving behavior can be analyzed by observing by the equations of motion in Section 2. Adjusting the CoG location changes the parameters, namely the static vertical load and the values of *a* and *b*. Such parameters influence the lateral side slip angles, and hence the lateral forces. By looking at Equation (8), it follows that shifting the CoG towards the front leads to an increase in the front side slip angle, which may lead to understeering as shown in Equation (13). Furthermore, Equation (3) leads to the same conclusion as at constant lateral forces and steering angle, increasing the value of *a* leads to a higher turning moment (i.e., oversteer). The same reasoning can be applied by looking at the understeer gradient in Equation (11), as it expresses the cornering behavior as a function of the static vertical load. Increasing the vertical front load (i.e., reducing the parameter *a*) leads to understeering, whereas decreasing it leads to oversteering. Such observations match with the results shown in Figure 10 corresponding to the linear region. Moreover, by applying Equation (12), the modeled value of the understeer gradient is calculated: the gradient has a value of approximately zero when loading the vehicle on the rear while it reaches a value of about 0.3 when loading the vehicle on the front. To model the dynamics at a higher speed more precisely, a more advanced model may be implemented; however, such observations show that the main dynamics of the vehicle are well represented by the bicycle model.

Regarding the longitudinal motion, Equation (20) shows that the force is dependent on the longitudinal slip, and linearly to the vertical force. Hence increasing the tire load leads to an increase in the maximum force assuming that the tire-road coefficient is constant. Furthermore, the tire model assumes no pitch motion, hence no load transfer while accelerating or decelerating. Given that load transfer increases at high acceleration and when the center of gravity is high, it is neglected for the scope of this research. However, implementing load transfer in the tire model may further improve the performance of the controller and of the vehicle for very high acceleration values [30]. Given the results shown in Section 3, it follows that the optimal location of the CoG is highly dependent on the application.

## 5. Conclusions

From this research, it follows that vehicle performance can be improved by implementing active safety systems such as a yaw rate controller or wheel slip controller. Such systems considerably increase handling, performance and stability as they allow the driver to maintain control of the vehicle, hence preventing accidents by reducing excessive over and understeer while cornering and increasing the stability while accelerating or braking. Moreover, we conclude that performance is highly related to loading conditions, where the weight distribution should be adjusted to meet the cornering requirements.

For a rear-wheel driven vehicle, locating the CoG rearward leads to a higher understeering in the linear tire region, whereas shifting it to the front reduces such behavior. However, such distribution has also an effect on the longitudinal dynamics with regard to braking and launching, as decreasing the vertical load on the driving axle decreases the maximum force that can be developed, hence decreasing the performance in terms of braking distance and acceleration time. Since excessive over- and understeering is often undesired, for a rear-wheel driving vehicle, a trade-off has to be achieved.

To conclude, weight distribution and control algorithms highly influence driving behavior in terms of stability, handling and hence, safety. This research has provided an insight on how control systems can be implemented on commercial vehicles with different weight distribution to increase their performance. For future research, we will further explore topics such as vehicle stability concerning road friction, saturated tire force, and advanced control (prescribed performance control and sliding mode control [33]) to further improve the vehicle driving performance.

## Figures and Tables

**Table 1:** Yaw rate controller parameters.

$K_{P}$	$K_{I}$
2	0.6

**Table 2:** Wheel slip controller parameters.

$K_{P}$	$K_{I}$
4	8

**Table 3:** Vertical static load on each axle; experiments on launch and cornering performance).

Experiment	$F_{z1}$ [N]	$F_{z2}$ [N]
Loaded front	92.9	69
Loaded rear	40.5	97.4
Unloaded	56.3	76.2

**Table 4:** Vertical static load on each axle braking experiment.

Experiment	$F_{z1}$ [N]	$F_{z2}$ [N]
Loaded front	89.9	71.4
Loaded rear	54.6	95.6
Unloaded	56.3	76.4

## Data Availability

The data presented in this study are available on request from the corresponding author.
